# Nivolumab-Induced PRES (Posterior Reversible Encephalopathy Syndrome)

**DOI:** 10.7759/cureus.40533

**Published:** 2023-06-16

**Authors:** Kanica Yashi, Jaswinder Virk, Taral Parikh

**Affiliations:** 1 Internal Medicine, Bassett Health Care, Cooperstown, USA; 2 Cardiology, Bassett Health Care, Cooperstown, USA; 3 Pediatrics, Hamilton Health Center, Harrisburg, USA

**Keywords:** radiologic pres, reversible posterior cerebral edema syndrome, immunotherapy, nivolumab, posterior reversible encephalopathy syndrome (pres)

## Abstract

PRES (posterior reversible encephalopathy syndrome) is a clinical-radiographic syndrome comprising clinical symptoms, including headache, vision abnormalities, nausea/vomiting, seizures, and posterior cerebral white matter edema seen as radiographic changes. Commonly, PRES is known to occur with severely elevated blood pressure, or eclampsia. However, in recent times, increasing cases of PRES have been observed in patients being treated with immunotherapy or chemotherapy. Nivolumab is an immunotherapy used in the treatment of various cancers and is an increasingly identified cause of PRES. A few case reports exist in the literature. We report a case of nivolumab-induced PRES.

## Introduction

PRES (posterior reversible encephalopathy syndrome) is a clinical-radiographic syndrome comprising a clinical syndrome of headache, confusion or decreased level of consciousness, vomiting, visual changes, and seizures and associated with characteristic neuroimaging findings of posterior cerebral white matter edema [1]. PRES is increasingly recognized in the medical literature. However, the incidence remains unknown. PRES is most commonly observed in medical conditions such as hypertensive encephalopathy, eclampsia, and cytotoxic and immunosuppressant drugs [2].

The name of this syndrome, PRES, is often misleading, as the syndrome is not always reversible and is often not confined to either the white matter or the posterior regions of the brain. Although PRES's pathogenesis remains unclear, it is thought to be due to arterial hypertension leading to cerebral autoregulatory failure, causing cerebral ischemia or endothelial dysfunction [3].

Recently, several cases of PRES due to immunotherapy drugs have been increasingly reported. One such drug that falls into this category is nivolumab. Nivolumab is an immunotherapy drug used to treat head, neck, lung, gastrointestinal, and several other tumors. It is a monoclonal antibody that targets the anti-PD1 receptor, an immune checkpoint. We present a case of PRES in a 74-year-old male who was receiving nivolumab to treat esophageal carcinoma [4,5]. 

## Case presentation

A 74-year-old male with a medical history significant for cardiomyopathy with an implantable cardiac defibrillator, hypertension, and esophageal cancer in partial remission presented to our hospital with four weeks of progressive vision loss, headache, and nausea. He reported seeing blurry images with on-and-off flashes of light. He had received six cycles of nivolumab infusion for esophageal cancer treatment. His last nivolumab infusions cycle was three weeks before his hospital presentation. Before this, he was also treated with 10 cycles of FOLFOX (folinic acid, fluorouracil, and oxaliplatin), which ended in January 2023, and since then, he has only been on nivolumab. For this ongoing vision issue, an ophthalmologist was consulted, and the eye examination did not reveal anything acutely worsening. However, a diagnosis of chronic amblyopia and cataracts was made, for which a six-month follow-up was recommended. However, the continued worsening of the patient's vision brought him to the hospital. His home medications included rosuvastatin and aspirin. On presentation, his vitals were stable. He had a temperature of 98.2 F, a pulse of 74, a blood pressure of 114/71, a respiratory rate of 18, and a pulse-oxygen saturation of 98% in room air (Table 1).

**Table 1:** Vital signs

Temperature	98.2 F
Pulse rate	74
Blood pressure	114/71
Respiratory rate	18
Oxygen saturation	98% on room air

On exam, he was alert and oriented and appeared cachectic with a BMI of 20.24 but not distressed. His cardiovascular, lung, and gastrointestinal exams were normal. His neurological exam indicated normal strength and sensation bilaterally in both upper and lower extremities. His reflexes were normal throughout. Cranial nerves 1-12 were grossly intact. Gait and balance were normal.

**Figure 1:**
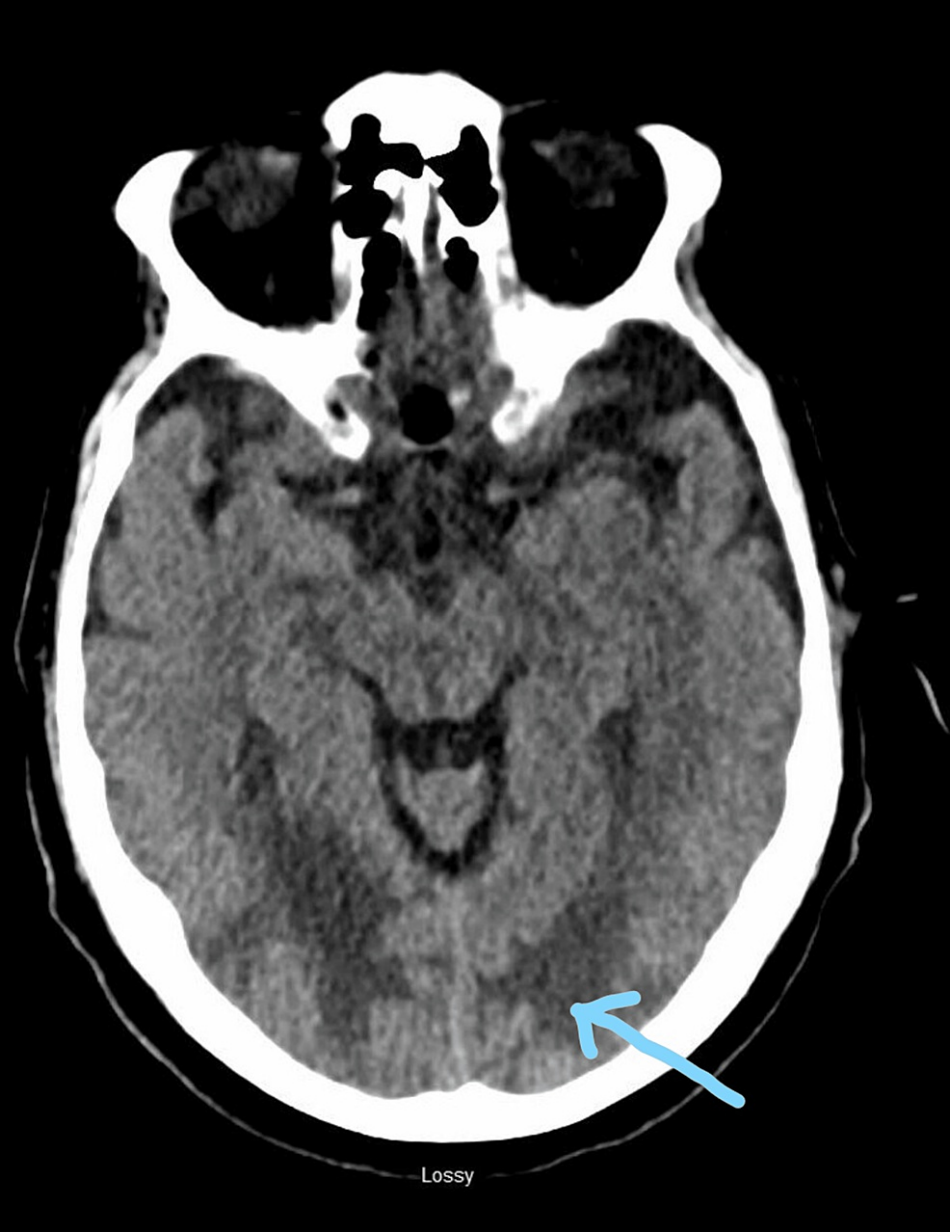
CT head of brain showing extensive hypodensity in bilateral occipital white matter most likely secondary to vasogenic edema (as marked by the arrow)

**Figure 2:**
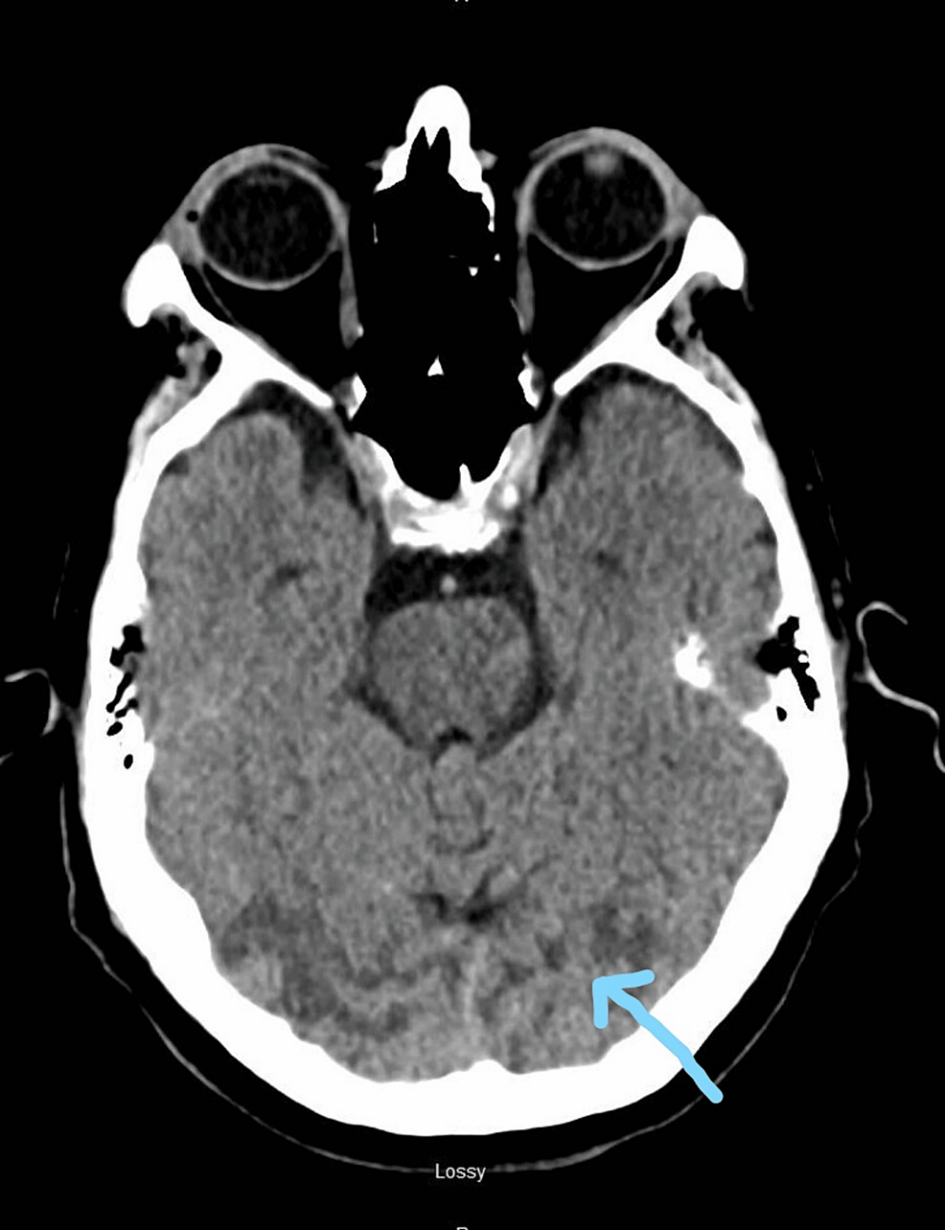
CT head of brain showing extensive hypodensity in bilateral occipital white matter most likely secondary to vasogenic edema (as marked by the arrow)

A CT head indicated extensive hypodensity secondary to vasogenic edema involving occipitoparietal regions and sparing the gray matter (Figures 1, 2). No acute infarction, hemorrhage, or midline shift was noted on the CT. An MRI of the brain was recommended for further investigation, which unfortunately could not be pursued due to the implantable cardiac defibrillator in the patient.

Neurology was consulted, who suggested that the patient has PRES likely due to nivolumab use and recommended starting the patient on dexamethasone 4 mg twice daily for 15 days for treatment. A recommendation to stop nivolumab indefinitely was made with a repeat CT head scan in two weeks for follow-up. The patient was started on treatment with dexamethasone and observed in the hospital for three days with close monitoring of his vitals and neurochecks. His blood pressure was never noted to be high while in the hospital. He continued to be stable during his admission to the hospital and noticed some improvement in his vision by day three. Neurology and physical therapy cleared the patient, and he was discharged home on oral dexamethasone for 11 more days. An outpatient follow-up with neurology and oncology was arranged. Nivolumab was stopped indefinitely.

Two weeks later, a brain CT scan showed improvement in the occipitoparietal region's vasogenic edema and the patient's vision.

## Discussion

PRES is a clinical-radiographic syndrome characterized by neuroimaging findings of posterior cerebral white matter edema and clinical symptoms of headache, confusion or decreased level of consciousness, vomiting, visual changes, and seizures. Due to the absence of well-defined criteria for diagnosing PRES, the condition is often underdiagnosed, and the pathophysiology needs to be better understood. One such theory postulated with severe arterial hypertension, such as in patients with hypertensive emergencies or pre-eclampsia, is that cerebral hyperperfusion leads to vascular leak, breaking the blood-brain barrier and thus causing vasogenic edema [3,4].

However, not all cases of PRES occur in patients with high blood pressure. Interestingly, PRES has been observed in normotensive patients as well. These cases were typically observed in patients receiving immunotherapy, chemotherapy, sepsis, or autoimmune disorders. This has been postulated to result from circulating endogenous or exogenous toxins in these conditions altering endothelial dysfunction, causing hypoperfusion and vasoconstriction that disrupt the blood-brain barrier. Such was the case in our patient, who developed PRES likely due to using nivolumab as immunotherapy and was normotensive throughout [6-8].

Sepsis and autoimmune disorders can directly affect the blood-brain barrier due to neuroinflammation, causing intravascular fluid extravasation and edema [9-11].

Identifying the etiology and management of PRES can be essential to preventing life-threatening complications. In cases where high blood pressure is noted to be the etiology, typically a systolic above 160, the goal is to treat the pressure to keep it between 130 and 150 mmHg systolic and 80 and 100 mmHg diastolic. If PRES is attributed to immunotherapy or chemotherapy, the recommendation is to stop or reduce the dose of the medication. Treating the underlying etiology is recommended in cases of sepsis or autoimmune disorders [12-15].

A timely diagnosis and treatment of PRES results in a favorable prognosis. Most symptoms, especially visual ones, typically resolve in a few days or weeks. Although, in a few cases, the visual deficits have remained or partially improved.

## Conclusions

PRES is an underrecognized clinical-radiographic syndrome that can have severe and poor outcomes if not treated timely. However, a timely diagnosis and treatment can lead to favorable outcomes. Elevated arterial blood pressure is a known common etiology of PRES; however, an important point to note would be other causes of PRES, such as immunotherapeutic agents, chemotherapeutic agents, sepsis, and autoimmune conditions. Knowledge about this syndrome and communication among various medical departments can be instrumental in its early diagnosis and treatment.
